# Preparation and Characterization of Fe_3_O_4_/Poly(HEMA-co-IA) Magnetic Hydrogels for Removal of Methylene Blue from Aqueous Solution

**DOI:** 10.3390/gels10010015

**Published:** 2023-12-22

**Authors:** Michael A. Ludeña, Flor de L. Meza, Reneé I. Huamán, Ana M. Lechuga, Ana C. Valderrama

**Affiliations:** 1 Laboratorio de Investigación en Biopolímeros y Metalofármacos (LIBIPMET), Facultad de Ciencias, Universidad Nacional de Ingeniería, Av. Tupac Amaru 210, Rimac, Lima 15333, Peru; michael.ludena.h@uni.pe (M.A.L.); renee.huaman.q@uni.pe (R.I.H.); 2 Tecnología Materiales para Remediación Ambiental (TecMARA), Facultad de Ciencias, Universidad Nacional de Ingeniería, Av. Tupac Amaru 210, Rimac, Lima 15333, Peru; flor.meza.l@uni.pe; 3 Departamento Académico de Química, Facultad de Ciencias Químicas, Físicas y Matematicas, Universidad Nacional de San Antonio Abad del Cusco (UNSAAC), Av. de la Cultura 733, Cusco 921, Peru; ana.lechuga@unsaac.edu.pe

**Keywords:** magnetic, hydrogel, adsorption, methylene blue, composite, removal

## Abstract

In the present study, Fe_3_O_4_/poly(2-hydroxyethyl methacrylate-co-itaconic acid) magnetic hydrogels (MHGs) were prepared by in situ synthesis of Fe_3_O_4_ magnetic particles in hydrogels (HGs). The resulting magnetic hydrogels were characterized by Fourier transform infrared spectroscopy (FT-IR), X-ray diffraction (XRD), thermogravimetric analysis (TGA), a vibrating sample magnetometer (VSM), scanning electron microscopy (SEM), and N_2_ adsorption–desorption. The effect of Fe_3_O_4_ on the swelling behavior and adsorption of methylene blue (MB) dye of the prepared hydrogel was studied. Parameters such as the dose, pH, contact time, and MB initial concentration were investigated. The results show that 75% (HG) and 91% (MHG) of MB (200 mg/L) were removed at doses of 2 mg/mL and 1 mg/mL, respectively, under a pH of 6.8 and a contact time of 10 min. The adsorption behavior followed the Langmuir isotherm model, indicating that the adsorption process takes place in monolayers and on homogeneous surfaces. The Langmuir capacities for MB adsorption using the HGs and MHGs were 78 and 174 mg/g, respectively. The adsorption kinetics followed a pseudo-second-order kinetic model. In addition, thermodynamic studies carried out show that the adsorption process is spontaneous and endothermic. Adsorption–desorption studies indicate that the magnetic hydrogel can remove MB for four cycles with removal efficiencies above 90%. Therefore, a MHG is suitable as an alternative material for MB adsorption.

## 1. Introduction

It is not a novelty that for several years, industrial activities have been the cause of environmental problems as a result of the contamination of water bodies with various pollutants, such as plastics, heavy metals, dyes, etc. [1,2,3]. In particular, the textile industry is one of the main polluters of the environment due to the discharge of large quantities of wastewater with a high concentration of different types of dyes [4,5,6]. Among these, methylene blue (MB) is the most widely used cationic dye for dyeing wood, cotton, and silk [7]. However, MB is toxic, and the health risks associated with this dye may include gastrointestinal, respiratory, central nervous system, cardiovascular, and dermatological complications, among others [8]. In addition, MB has a high molar absorption coefficient, which can cause attenuation of sunlight transmittance, affecting the photosynthesis process, chemical oxygen demand (COD), biological oxygen demand (BOD), and oxygen demand levels, disrupting the entire aquatic ecosystem [9]. All these problems associated with MB make it necessary to assess or evaluate the different technologies available to remove MB from wastewater.

Several methods, such as coagulation [10], membrane filtration [11], photodegradation [12], and adsorption [13], have been used to remove dyes from wastewater. Among all these methods, the adsorption technique is usually considered efficient, non-toxic, environmentally friendly, and simple to remove pollutants from wastewater [14]. For this reason, in the last years, various materials such as activated carbon, mineral clay [15], metal oxide particles [16], graphene oxide [17], silica [18,19,20], zeolites [21,22], hydrogels [23,24], etc., have been used in recent years for the removal of dyes from wastewater by the application adsorption technique. Hydrogels are crosslinked polymers with three-dimensional networks, which are widely used to remove environmental pollutants, and unlike other adsorbents, hydrogels adsorb pollutants in a highly porous network [25]. An emerging approach to impart new properties to polymeric hydrogels, or to improve their adsorption capacity, is to combine them with other materials to form composite materials. A wide range of materials, such as carbon-based nanomaterials (graphene sheets, graphene oxide sheets, carbon nanotubes), ceramic inorganic particles (hydroxyapatite, silicates), metal particles (gold, silver), and metal oxide particles (magnetite, hematite) can be integrated into hydrogel networks to form these composites, also known as hybrid hydrogels [26]. The introduction of magnetic particles such as Fe_3_O_4_ in the synthesis of adsorbents has led to an improvement in the adsorption capacity of these materials due to the high surface area and active sites. In addition, Fe_3_O_4_ particles have the advantage of being biocompatible and having a low toxicity [27,28]. On the other hand, the affinity of the magnetic particles to an external magnetic field allows them to be easily and quickly separated from the aqueous solution [29]. Therefore, the use of Fe_3_O_4_ magnetic particles to modify other adsorbents such as hydrogels can improve their adsorption capacity. Mehdizadeh et al. [30] presented the synthesis of a new magnetic nanocomposite for the removal of MB dye from aqueous solutions. First, they synthesized magnetite nanoparticles using a co-precipitation method and then modified them with 3-aminopropyltriethoxysilane (APTES) and acryloyl chloride. Then, they grafted itaconic acid (IA) and 2-hydroxyethyl methacrylate (HEMA) onto the modified nanoparticles by in situ copolymerization to prepare the final nanocomposite. The obtained nanocomposite can remove the dye by more than 89%. On the other hand, Beigi et al. [31] performed the synthesis of magnetic hydrogels based on pectin and the ex situ addition of Fe_3_O_4_ magnetic nanoparticles; the potential of the material for adsorption of organophosphorus chlorpyrifos, pesticide, and crystal violet organic dye was investigated. In contrast, Singh et al. [32] reported the synthesis of magnetic hydrogels based on carboxymethyl cellulose by in situ mineralization of Fe^2+^/Fe^3+^ ions in the hydrogel network, for which they first performed the synthesis of a carboxymethyl cellulose hydrogel. Subsequently, Fe_3_O_4_ nanoparticles were formed within the hydrogel matrix by the co-precipitation method. The prepared adsorbents were used for the removal of methylene blue (MB) from aqueous solutions.

In the present study, magnetic hydrogels based on Fe_3_O_4_/poly(HEMA-co-IA) were prepared for the first time and investigated for the removal of MB dye from aqueous solutions. For this purpose, poly(HEMA-co-IA) hydrogels were first prepared by radical copolymerization of 2-hydroxyethyl methacrylate (HEMA), itaconic acid (IA), and the crosslinker N,N′-methylenebisacrylamide (MBA). Ammonium persulfate (APS) together with N,N,N′,N′-tetramethylethylenediamine (TEMED) were used as initiator and accelerator, respectively. The magnetic hydrogels were synthesized in situ by incorporating Fe^2+^ and Fe^3+^ ions into the poly(HEMA-co-IA) hydrogels and then immersed in a NaOH solution. The properties of these adsorbents were characterized by various analyses, such as Fourier transform infrared spectroscopy (FT-IR), X-ray diffraction (XRD), thermogravimetric analysis (TGA), scanning electron microscopy (SEM), a vibrating sample magnetometer (VSM), and N_2_ adsorption–desorption. In the adsorption experiment, the effect of the pH, dosage, contact time, and MB initial concentration were investigated. Kinetic, isothermal, and thermodynamic studies were also performed to evaluate the type of adsorption processes. In addition, the reusability of the synthesized adsorbent was evaluated with sequential MB adsorption–desorption cycles.

## 2. Results and Discussion

### 2.1. Synthesis

**Figure 1:**
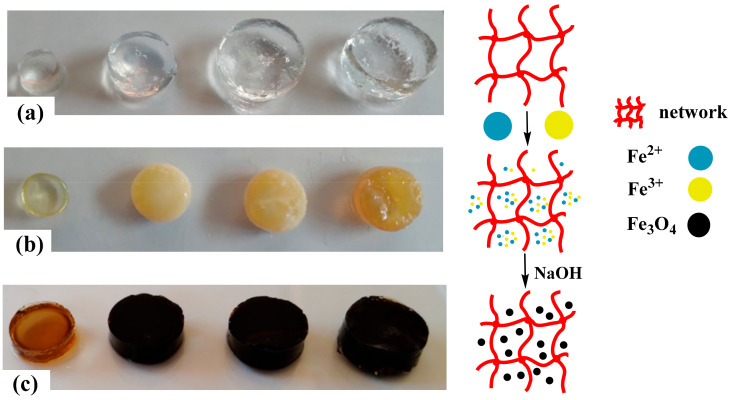
Preparation process of the magnetic hydrogels. (**a**) Hydrogels swollen in water for 24 h (from left to right, we have hydrogels HG-0, HG-5, HG-7.5, and HG-10), (**b**) hydrogels loaded with Fe^2+^ and Fe^3+^ cations, and (**c**) appearance of the hydrogels when the Fe_3_O_4_ magnetic particles have been formed in situ (from left to right we have hydrogels MHG-0, MHG-5, MHG-7.5, and MHG-10).

The porous structure of the hydrogels and the presence of carboxyl groups (–COO^−^) in the main chains allow easy binding to Fe^2+^ and Fe^3+^ cations via electrostatic interactions. Figure 1 shows a schematic representation of the formation of the magnetic hydrogels. Figure 1a shows the image of the water-swollen hydrogels. The incorporation of Fe^2+^ and Fe^3+^ changes the color of the swollen hydrogel to yellow-orange, and the color intensity is related to the amount of IA in the hydrogel; i.e., the higher the number of carboxylate groups (–COO^−^), the higher the concentration of Fe^2+^ and Fe^3+^ cations (Figure 1b). When hydrogels loaded with metal ions are transferred to the sodium hydroxide solution, a rapid color change from yellow to black occurs. This indicates the formation of Fe_3_O_4_ particles inside the hydrogel [33,34] (Figure 1c).

### 2.2. The Characterization of the Adsorbents

**Figure 2:**
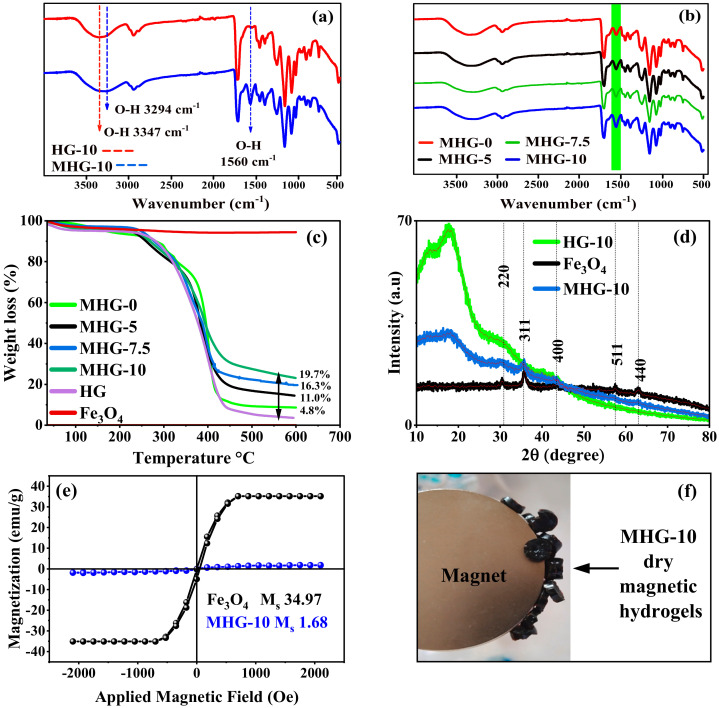
FT-IR spectra (**a**) HG-10 and MHG-10, (**b**) magnetic hydrogels with different amounts of IA, (**c**) TGA curves of Fe_3_O_4_, HGs, and MHGs, (**d**) X-ray diffractograms of Fe_3_O_4_, HG-10, and MHG-10, (**e**) magnetization hysteresis loops of the Fe_3_O_4_ and MHG-10, and (**f**) image of the dry MHG-10 magnetic hydrogels attracted by the magnet.

According to the FT-IR study, the Fe_3_O_4_ particles have three characteristic signals in the infrared spectrum and appear around 579, 1623, and 3410 cm^−1^, which correspond to the Fe-O stretching, O-H deformation, and O-H stretching vibrations, respectively [35]. These last two vibrations correspond to the hydroxyl groups on the surface of the Fe_3_O_4_ particles [36]. The FT-IR spectra of HG-10 and MHG-10 are shown in Figure 2a; the O–H stretching vibration signal of the hydroxyl groups on the surface of the Fe_3_O_4_ particles was shifted to 3294 cm^−1^ due to electrostatic interaction with the –COOH, –COO^−^, -OH groups [33]. For the same reason, the O–H deformation vibration signal of the hydroxyl groups was shifted to 1560 cm^−1^. Figure 2b shows the FT-IR spectra of magnetic hydrogels with different amounts of IA (MHG-0, MHG-5, MHG-7.5, and MHG-10), and it is observed that the intensity of the signal at 1560 cm^−1^ increases, indicating that the amount of magnetic particles in the MHG-10 magnetic hydrogel is higher. This increase in intensity is directly related to the amount of IA; i.e., as the amount of IA in the hydrogels increases, the amount of magnetic particles in the hydrogels also increases.

According to the TGA study, the amount of Fe_3_O_4_ in the magnetic hydrogels was determined by thermogravimetry. Figure 2c shows the thermograms of the MHG, HG, and bare Fe_3_O_4_ particles. In contrast to the Fe_3_O_4_ particles, the HGs and MHGs underwent a thermal degradation between 300 and 400 °C of the organic part, i.e., the poly(HEMA-co-IA). The difference in the residual weight percentage corresponds to the Fe_3_O_4_ particles. Therefore, the amount of Fe_3_O_4_ particles in hydrogels MHG-0, MHG-5, MHG-7.5, and MHG-10 is 4.8%, 11.0%, 16.3%, and 19.7%, respectively, as shown in Figure 2c. These results confirm that the amount of IA has a direct effect on the amount of Fe_3_O_4_ particles to be formed in the hydrogel matrix.

According to the XRD study, X-ray diffraction patterns for the synthesized bare iron oxide Fe_3_O_4_ particles, hydrogel, and magnetic hydrogel are shown in Figure 2d. Five characteristic peaks for bare Fe_3_O_4_ at 30.34 (220), 35.79 (311), 43.62 (400), 57.54 (511), and 62.93 (440) indicate that the obtained particles are magnetite with an inverse spinel structure [37]. The MHG-10 patterns also show similar characteristic peaks of the magnetite particles. These results indicate that the crystalline structure of magnetite is not altered by synthesis in the hydrogel network. The X-ray diffractogram of the hydrogel shows a common broad diffraction peak at around 2θ = 10°–25°, which is due to the amorphous nature of the HG-10 hydrogel [38]. This is in agreement with the results reported by Sivudu and Rhee [34].

According to the VSM study, the magnetic properties of the MHG-10 hydrogel and Fe_3_O_4_ particles synthesized by the chemical co-precipitation method for comparison purposes were analyzed with a VSM, and the result is shown in Figure 2e. The magnetic saturation of the synthesized MHG-10 magnetic hydrogel and Fe_3_O_4_ was found to be 1.68 and 34.97 emu/g, respectively. The decrease in the magnetic saturation value is due to the presence of the diamagnetic poly(HEMA-IA) hydrogel. However, this MHG-10 hydrogel still exhibits magnetic properties, as shown in Figure 2f. A similar magnetic saturation value of 1.66 emu/g was obtained for the Fe_3_O_4_/SiO_2_/poly(NIPAM-co-DMA) composite hydrogel [39] and 3.2 emu/g for the Fe_3_O_4_@SiO_2_-chitosan hydrogel [40].

**Figure 3:**
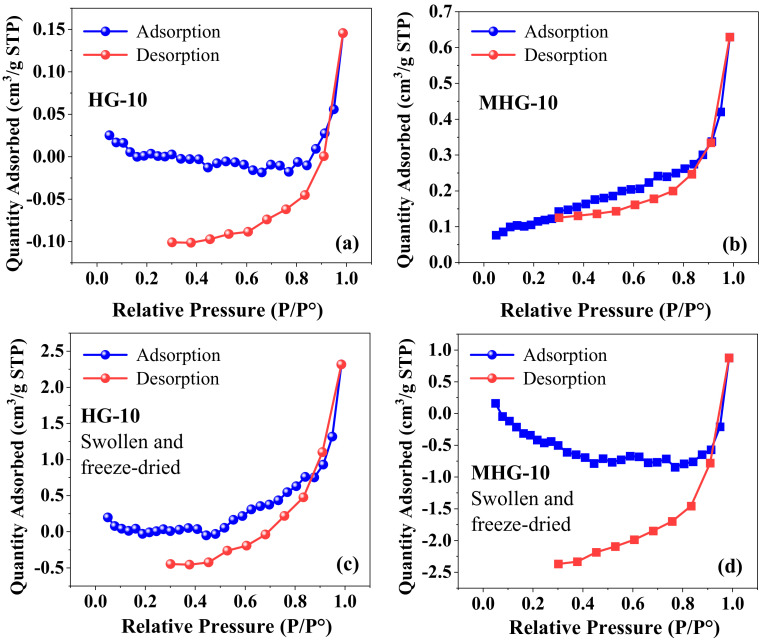
Adsorption–desorption isotherms of dry (**a**,**b**) and swollen-water/freeze-dried (**c**,**d**) hydrogels.

In the N_2_ adsorption–desorption study, the nitrogen adsorption–desorption isotherm of dry hydrogels HG-10 and MHG-10 is shown in Figure 3a,b. According to the IUPAC classification, the isotherm of the hydrogels is type III; i.e., there is no discernible monolayer formation; adsorbate–adsorbate interactions are relatively weak, and the adsorbed molecules cluster around the most favorable sites on the surface of the solid [41]. The BET surface area of the HG-10 hydrogel was 0.0008 m^2^/g, while that of the MHG-10 magnetic hydrogel was 0.4240 m^2^/g. The pore volume also increases from 0.00022 cm^3^/g to 0.00094 cm^3^/g for HG-10 and MHG-10, respectively. The increase in surface area and pore volume is attributed to the introduction of Fe_3_O_4_ into the internal structure of the HG-10 hydrogel. Similar values were reported for the chitosan composite with vanadium–titanium magnetic particles CS-VTM-2.0 (surface area = 0.335 m^2^/g and pore volume = 0.0008 cm^3^/g) [42]. Figure 3c and d show the adsorption–desorption isotherm of HG-10 and MHG-10 hydrogels swollen in water for 24 h and then freeze-dried. Similar to the dried hydrogel sample HG-10 (Figure 3a), the hystereses loop does not close at low pressure. Yue et al. [43] mention four reasons for this phenomenon. First, pore deformation is caused by the adsorption of liquid nitrogen or filler. Second, some liquid nitrogen molecules cannot be released. Third, nitrogen molecules are trapped by the adsorption potential. Fourth, the existence of ink-bottle pores in the sample can cause this situation. There are research papers that report this form of hysteresis, such as Li et al. [44] and Qi et al. [45], but they do not give a precise reason for this phenomenon.

**Figure 4:**
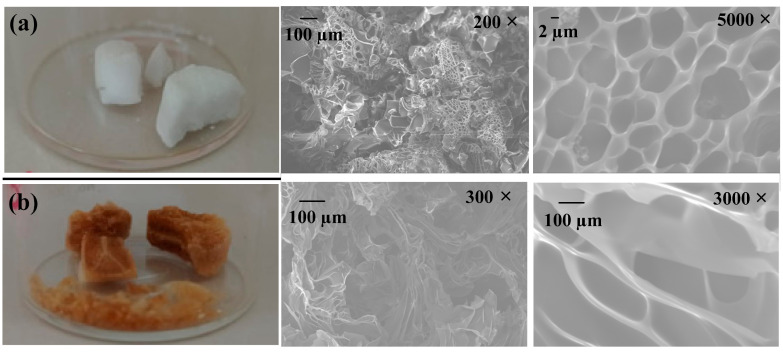
SEM images of (**a**) HG-10 and (**b**) MHG-10 hydrogels swollen in water for 24 h at RT.

In the SEM study, the SEM micrograph reveals microstructural features of HG-10 and MHG-10 hydrogels swollen in water for 24 h at RT and dried by freeze-drying (Figure 4). The SEM image of the HG-10 hydrogel shows a highly porous and homogeneous microstructure (Figure 4a). The microstructure of the MHG-10 magnetic hydrogel was affected by the formation of Fe_3_O_4_ particles within the hydrogel matrix (Figure 4b). These SEM images show that the Fe_3_O_4_ particles lead to a less homogeneous microstructure, and the pores are less interconnected.

### 2.3. Degree of Swelling of the HGs and MHGs

The results of the swelling degree (q) are shown in Table 1. Compared with the HGs, the swelling values of hydrogels loaded with Fe_3_O_4_ particles are higher. The same swelling behavior was observed in the polyacrylamide magnetic hydrogels prepared by Sivudu and Rhee [34]. However, when the magnetic hydrogels were prepared with 2-acrylamide-2-methyl-1-propanesulfonic acid, the swelling behavior was the opposite [46]. According to the authors, this behavior is due to the fact that the Fe_3_O_4_ particles act as knots binding the polymer chains, thus limiting the expansion of the magnetic hydrogels. However, several factors must be taken into account; for example, the formation of Fe_3_O_4_ particles with sodium or ammonium hydroxide causes the deprotonation of the carboxyl groups of IA, which favors the swelling of the hydrogels, since the carboxylates (–COO^−^) exert a repulsive force, causing the polymer chains to expand.

Another factor that could positively contribute to the swelling is the presence of hydroxyl groups on the surface of the Fe_3_O_4_ particles. On the other hand, interactions between functional groups of the hydrogel polymer chains with Fe_3_O_4_ particles could negatively contribute to the swelling. However, these are hypotheses that need to be investigated for a better understanding of the effect of Fe_3_O_4_ particles in hydrogels. Swelling experiments were also performed in a 0.01 mol/L sodium hydroxide solution and a 0.01 mol/L hydrochloric acid solution. Under acidic conditions, little swelling occurs in non-magnetic and magnetic hydrogels because deprotonation of the carboxyl groups of IA does not occur, which favors the formation of hydrogen bonds between the polymer chains of the hydrogel, which hinders swelling. Under basic conditions, the deprotonation of the carboxyl groups and the repulsion of the carboxylate ions favor the expansion of the hydrogel (Table 1). The swelling of the magnetic hydrogels is higher in water than in a NaOH solution, possibly due to the higher interaction strength between the hydroxyl groups (–OH) on the surface of the particles and the carboxylate groups (–COO^−^), which hinders swelling.

### 2.4. Methyl Blue Adsorption

Previously, some MB adsorption experiments were performed with dry hydrogels and hydrogels swollen in distilled water for 24 h. It was observed that MB adsorption was better when performed with hydrogels previously swollen in distilled water.

**Figure 5:**
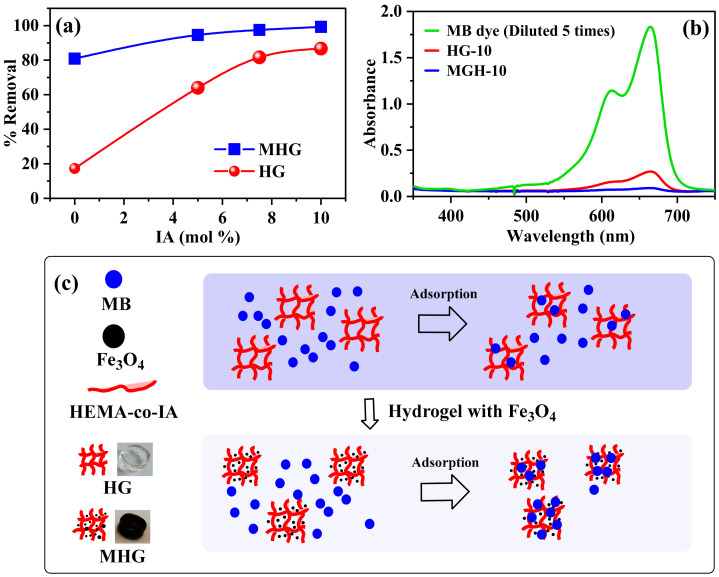
(**a**) Removal percentage of HGs and MHGs (V = 10 mL, C_0_ = 50 mg/L, adsorbent dose = 10 mg, contact time = 10 min, pH = 6.8, and RT), (**b**) UV–VIS spectra after MB removal using HG-10 and MHG-10 hydrogels, and (**c**) scheme of MB adsorption with HG-10 and MHG-10.

The removal percentage of all prepared HGs and MHGs was examined, and the results are shown in Figure 5. In Figure 5a, it can be seen that the removal with homopolymer hydrogels is low due to their non-ionic character. The removal percentage of the HGs increases with increasing IA content in the polymer chains due to the electrostatic interaction between the negative charge of the carboxylate groups (–COO^−^) of IA and the positive charge (=N^+^ −(CH_3_)_2_) of methylene blue [47]. It can also be observed that the removal percentage of the MHGs is higher than that of the non-magnetic hydrogels. This is due to the electrostatic interaction mentioned above. In addition, Fe_3_O_4_ particles can also adsorb dyes on their surface via van der Waals forces [48]. It can be concluded that the high adsorption capacity of the magnetic hydrogels is due to electrostatic and van der Waals interactions. Figure 5b is a representative UV–VIS spectra that show the successful removal of MB within 10 min. In addition, it can be seen in Figure 5b that the MB removal is by adsorption and not by degradation or scavengers. Figure 5c shows a schematic representation of MB adsorption with HG-10 and MHG-10.

Based on these experiments, the magnetic hydrogel prepared with 10 mol% IA (MHG-10) was selected for the remainder of this study, and to understand the effect of the incorporation of Fe_3_O_4_ particles in the hydrogel on the adsorption capacity (Q), the non-magnetic hydrogel HG-10 was also studied.

#### 2.4.1. Effect of pH

**Figure 6:**
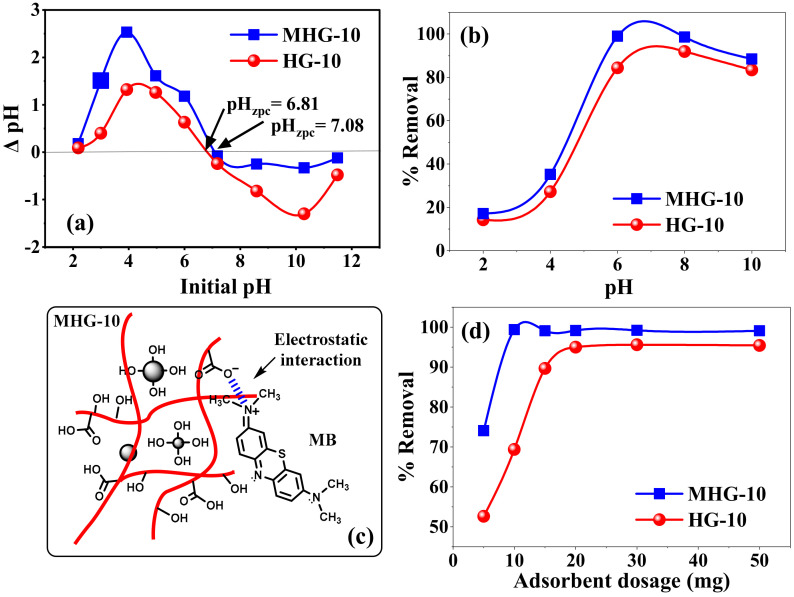
(**a**) Zero-point charge (ZPC) value of HG-10 and MHG-10 hydrogels, (**b**) effect of pH on the removal percentage of HG-10 and MHG-10 hydrogels (V = 10 mL, C_0_ = 50 mg/L, adsorbent dose = 10 mg, contact time = 10 min, and RT), (**c**) electrostatic interaction between MB and MHG-10, and (**d**) adsorbent dose (V = 10 mL, C_0_ = 50 mg/L, pH = 6.8, contact time = 10 min, and RT).

The initial pH of the solution can affect the surface charge of the adsorbent material and also control the electrostatic interaction between the adsorbent and the adsorbate. Therefore, it is an essential parameter for the removal of organic and inorganic adsorbates [49]. The effect of the initial pH value on the pHzpc and MB removal percentages of HG-10 and MHG-10 is shown in Figure 6a and b, respectively. The pHzpc values were found to be 6.81 and 7.08 for HG-10 and MHG-10, respectively (Figure 6a). According to the results, at pH > pHzpc, the surface of the adsorbents will have negative charges, and at pH < pHzpc, the surface of the adsorbents will have positive charges. The increase in negatively charged sites at a higher pH indicates the deprotonation of the carboxylic groups (-COOH) and the increase in the carboxylate groups (–COO^−^) on the adsorbent, which in turn explains the higher MB removal efficiency [50].

Figure 6b shows the effect of the initial pH on the removal percentage of HG-10 and MHG-10 for MB removal. The removal percentage varied with the initial pH of the solution. In an acidic medium, the interaction with the MB dye is not favorable because the carboxyl groups of IA are protonated (–COOH). At a low pH (pH < pHzpc), excess protons (H^+^) compete with the MB dye for adsorption sites on the adsorbent, and electrostatic repulsion also occurs between the two since they have an overall positive charge [51]. In addition, the formation of hydrogen bonds between the polymer chains occurs, preventing the expansion of the hydrogel, which hinders the diffusion of MB molecules within the hydrogel. At a high pH (pH > pHzpc), carboxyl groups of IA become carboxylate (–COO^−^) due to the presence of OH^-^. The adsorption sites in hydrogels become negatively charged, and electrostatic attraction (due to their opposite charge) between MB and the adsorbent leads to an increase in the removal percentage. The negative charge of the adsorbent also generates electrostatic repulsion between the adjacent ionized groups, inducing the expansion of the hydrogel and allowing easy diffusion of the MB within the hydrogel [52]. It should be noted that at pH = 10, the efficiency of the removal percentage decreases, which may be due to an increase in the concentration of ions in the solution, which compete with the adsorbent sites. The removal percentage of MHG-10 is higher than that of HG-10 due to the high surface area and active groups of the Fe_3_O_4_ particle. These results show that one of the main mechanisms of adsorption of the MB dye on the hydrogels is the electrostatic interaction, see Figure 6c.

#### 2.4.2. Effect of the Adsorbent Dose

The effect of the dosage of the adsorbent (5 to 50 mg) on the percentage removal efficiency of the MB dye on the HG-10 and MHG-10 hydrogels is shown in Figure 6d. According to the results in Figure 6d, it can be observed that by increasing the dosage of MHG-10 adsorbent from 5 to 10 mg, the removal efficiency increases from 74 to 99%. The increase in the removal efficiency by increasing the dose from 5 to 10 mg can be explained by the increase in the active sites of the adsorbent. However, at doses higher than 10 mg, the number of active sites is greater than the number of MB dye molecules. For the HG-10 adsorbent from 5 to 20 mg, the percentage removal efficiency increases from 52 to 94%, and a higher amount of adsorbent does not change the removal efficiency. Therefore, the optimum doses of MHG-10 and HG-10 adsorbents were selected to be 10 mg and 20 mg, respectively.

#### 2.4.3. Effect of Contact Time

**Figure 7:**
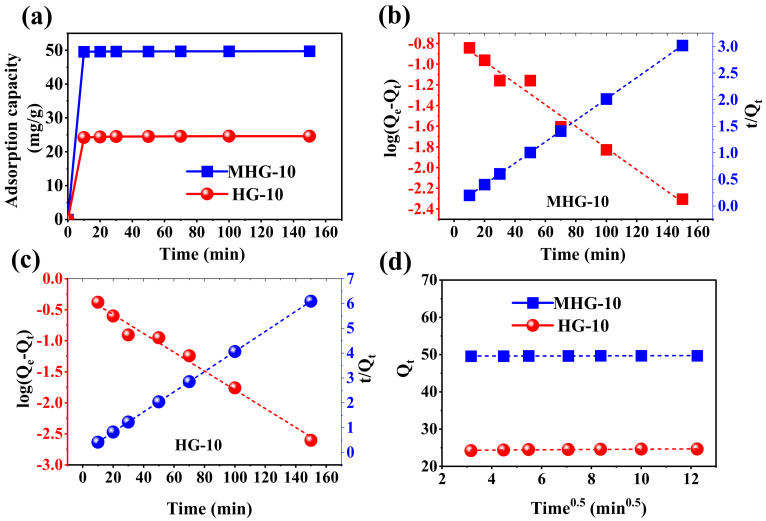
(**a**) Plot of the adsorption capacity as a function of time for the removal of MB with HG-10 and MHG-10 hydrogels (V = 10 mL, C_0_ = 50 mg/L, MHG-10 adsorbent dose = 10 mg, HG-10 adsorbent dose = 20 mg, pH = 6.8, and RT), (**b**,**c**) linear relationship of pseudo-first-order and pseudo-second-order kinetic models for MHG-10 and HG-10, respectively, and (**d**) linear relationship of the intraparticle diffusion kinetic model for MHG-10 and HG-10.

The adsorption capacity as a function of time was investigated using HG-10 and MHG-10. As shown in Figure 7a, HG-10 and MHG-10 rapidly adsorbed MB dye during the first 10 min, which may be due to the presence of high accessibility of empty active adsorption sites. The percentage of elimination achieved with MHG-10 during the first 10 min was 99.13%. From 10 to 150 min, the elimination was 99.40%, which is not a significant increase. For HG-10, the percentage of elimination in the first 10 min was 96.8%, and from 10 to 150 min, the elimination increased to 98.5%. On this basis, the contact time was set at 10 min.

The kinetic study of the adsorption process was Investigated using pseudo-first-order and pseudo-second-order kinetic models [29,53]. The linear form of the models is described by Equations (1) and (2):

Pseudo-first-order:

(1) $$\log\;\left(Q_{e}-{\;Q}_{t}\right)={\mathrm{logQ}}_{e}-\frac{K_{1}t}{2.303}\;$$

Pseudo-second-order:

(2) $$\frac{t}{Q_{t}}=\frac{1}{K_{2}Q_{e}^{2}}+\frac{t}{Q_{e}}\;$$

where Q_e_ (mg/g) and Q_t_ (mg/g) are the amount of MB adsorbed at equilibrium and at a given time (t, min), respectively. K_1_ (1/min) and K_2_ (g/mg·min) are the adsorption rate constants associated with the pseudo-first-order and pseudo-second-order, respectively. A linearized plot of log(Q_e_ − Q_t_) versus t gives the K_1_ and Q_e_ values, while a linearized plot t/Q_t_ versus t gives K_2_ and Q_e_. The results are shown in Figure 7b,c as well as in Table 2.

In the adsorption process, the pseudo-first-order and pseudo-second-order kinetic models are widely used; however, these models do not explain the diffusion mechanism well [50]. Therefore, the intraparticle diffusion model was also studied. The linear form of the model is described by Equation (3):

Intraparticle diffusion:

(3) $$Q_{t}=K_{D}t^{1/2}+C$$

where K_D_ (mg/g·min^1/2^) is the adsorption rate constant associated with the intraparticle diffusion, and C (mg/g) is the intercept, representing the thickness of the boundary layer. A linearized plot of Q_t_ versus t^1/2^ gives the K_D_ and C values. If the linear regression curve passes through the origin (C = 0), then intraparticle diffusion is the step that controls the adsorption process. Otherwise, the adsorption process is controlled by other mechanisms [54]. The results are shown in Figure 7d and Table 2.

According to the correlation coefficient (R^2^), the adsorption of MB by HG-10 and MHG-10 hydrogels is best described by the pseudo-second-order kinetic model. Also, the Q_e_ values calculated from the pseudo-second-order kinetic model were 24.652 and 47.718 mg/g for HG-10 and MHG-10, respectively, which are very close to the experimentally calculated Q_e_ values. The Q_e_ values calculated from the pseudo-first-order kinetic model were 0.529 and 0.171 mg/g for HG-10 and MHG-10, respectively, which have a large difference from those calculated experimentally (Table 2). This confirms that the pseudo-second-order kinetic model describes the kinetic behavior of the adsorption process very well. The high degree of linearity of the pseudo-second-order kinetics suggests that the step that controls the adsorption rate can be attributed to the chemical process [55]. In addition, the mass transfer process takes place through the active sites. According to the results obtained for the intraparticle diffusion model, the linear regression curve does not pass through the origin, indicating that diffusion is not the mechanism involved in the MB adsorption process using HG-10 and MHG-10. It should also be noted that the intercept parameter, C, for the MB adsorption on the MHG-10 magnetic hydrogel has a higher value than for the HG-10 adsorbent, which is attributed to the fact that the rate-limiting phase has a higher contribution from surface adsorption [50].

#### 2.4.4. Effect of MB Initial Concentration

**Figure 8:**
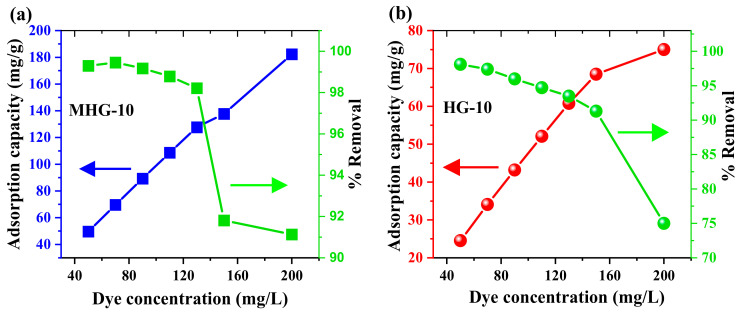
Plots of adsorption capacity and removal percentage as a function of MB initial concentration for (**a**) MHG-10 and (**b**) HG-10 adsorbents (V = 10 mL, C_0_ = MB initial concentration, contact time = 10 min, MHG-10 adsorbent dose = 10 mg, HG-10 adsorbent dose = 20 mg, pH = 6.8, and RT).

The effect of the MB initial concentration on the adsorption capacity of HG-10 and MHG-10 was studied. Figure 8 shows the plot of the adsorption capacity versus the MB initial concentrations of HG-10 and MHG-10. The results show that by increasing the initial concentration of MB dye from 50 to 200 mg/L, the removal percentage of MHG-10 (10 mg) decreases from 99.29 to 91.13%. However, the curve still does not reach a plateau, indicating that the active sites are not yet saturated. In the case of HG-10 (20 mg), the removal percentage decreases from 98.09 to 75.00%, and it can be seen that a plateau is beginning to be reached; i.e., the active sites are becoming saturated.

To understand the adsorption behavior, the data were inserted into Langmuir, Freundlich, Temkin, and Dubinin–Radushkevich (DR) adsorption models. The following Equations (4)–(7) represent the linear form of the isothermal models [29,54].

Langmuir:

(4) $$\frac{C_{e}}{Q_{e}}=\frac{1}{{\mathrm{bQ}}_{\max}}+\frac{C_{e}}{Q_{\max}},\;\;\;R_{L}=\frac{1}{1+{\;K}_{L}C_{O}}$$

Freundlich:

(5) $${\mathrm{logQ}}_{e}={\mathrm{logK}}_{F}+\frac{1}{n}\left({\mathrm{logC}}_{e}\right)\;$$

Temkin

(6) $$Q_{e}={\mathrm{Blnk}}_{T}-{\mathrm{BlnC}}_{e\;},\;B=\frac{\mathrm{RT}}{b_{T}}\;$$

Dubinin–Radushkevich

(7) $$\ln\left(Q_{e}\right)={\mathrm{lnQ}}_{s}-\beta \varepsilon^{2},\;\varepsilon =\mathrm{RTln}\left(1+\frac{1}{C_{e}}\right),\;E=\frac{1}{\sqrt{2\beta }}$$

where Q_e_ is the amount of adsorbed MB at equilibrium (mg/g), C_e_ is the concentration of MB at equilibrium (mg/L), Q_max_ is the maximum adsorption capacity (mg/g), K_L_ is the Langmuir isotherm constant (L/mg), which is associated with the adsorption energy, and K_F_ and n are the Freundlich isotherm constants that are related to the adsorption capacity (L/mg) and adsorption intensity. In the Temkin equation, K_T_ is the Temkin isotherm constant (L/g), b_T_ is the variation in the adsorption energy (J/mol), R is the universal gas constant (8.314 J/mol·K), and T is the temperature (K), and in the Dubinin–Radushkevich equation, Q_s_ is the theoretical isotherm saturation capacity (mg/g), β is the adsorption energy constant (mol/kJ)^2^, E is the adsorption energy (kJ/mol), and ε is related to the adsorption potential.

**Figure 9:**
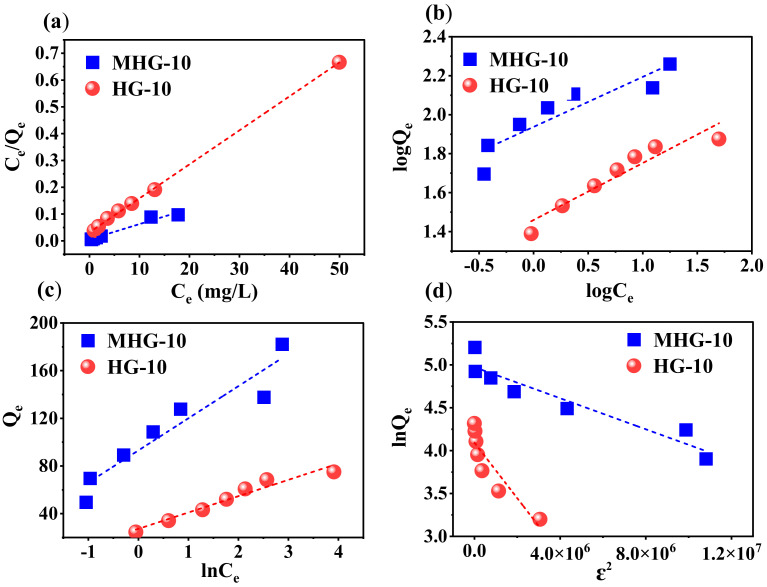
Plot of the linear relationship of the isothermal models (**a**) Langmuir, (**b**) Freundlich, (**c**) Temkin, and (**d**) Dubinin–Radushkevich for MHG-10 and HG-10.

Figure 9 shows the linear relationship of the isotherm models for MB adsorption by both adsorbents. The constants and parameters determined for the adsorption process are reported in Table 3. Based on these results, the experimental data obtained from the adsorption process best fit the Langmuir isotherm model (high R^2^ value in Table 4). Therefore, according to this Langmuir isotherm model, the adsorption process takes place in monolayers and on homogeneous surfaces [56]. The maximum adsorption capacity Q_max_ of the HG-10 and MHG-10 hydrogels for MB removal was determined to be 78.850 and 175.997 mg/g, respectively; the MHG-10 hydrogel has a higher adsorption capacity due to the Fe_3_O_4_ particles. In addition, the R_L_ parameter values were in the range of 0 and 1, indicating that the MB adsorption process was optimally performed. Due to the low R^2^ value, the other models do not reflect the equilibrium isotherm data of MB on MHG-10 and HG-10 well.

In Table 4, the adsorption capacity of MHG-10 for the removal of MB is compared with other adsorbents reported in the literature.

### 2.5. Thermodynamic Parameters

**Figure 10:**
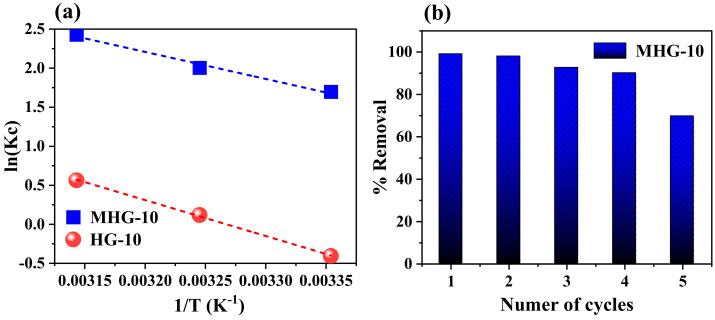
(**a**) Plot of ln(Kc) versus 1/T to determine thermodynamic parameters and (**b**) adsorption–desorption of methylene blue during five consecutive cycles. Adsorption conditions: dose 10 mg, initial concentration 50 mg/L, contact time 10 min, pH 6.8, and RT. Desorption conditions: 0.01 M HCl, 1 h contact time, and RT.

The thermodynamic parameters of enthalpy (ΔH^0^, kJ/mol), entropy (ΔS^0^, J/mol·K), and Gibbs free energy (ΔG^0^, kJ/mol) were used to determine whether the adsorption process was endothermic or exothermic. Equations (8)–(10) were used to determine the thermodynamic parameters [56]:

(8) $${\Delta G}^{0}=-\mathrm{RTln}\left(K_{C}\right)\;$$

(9) $${\Delta G}^{0}={\Delta H}^{0}-{T\Delta S}^{0}\;$$

(10) $$\ln\left(K_{C}\right)=\ln\frac{Q_{e}}{C_{e}}=\frac{-{\Delta H}^{0}}{\mathrm{RT}}+\frac{{\Delta S}^{0}}{R}\;$$

where K_c_ is the equilibrium constant, C_e_ is the MB concentration at equilibrium (mg/L), Q_e_ is the adsorption at equilibrium (mg/g), T is the absolute temperature (K), and R is the universal gas constant (8.314 J/mol·K). The values of ΔH^0^ and ΔS^0^ were obtained from the slope and intercept of the plot of ln(K_c_) versus 1/T (Figure 10a) and tabulated under Table 5. The negative ΔG^0^ value indicates that the adsorption process of MB dye using HG-10 and MHG-10 is spontaneous. Also, the negative ΔG^0^ value increases with an increasing temperature (from 25 to 45 °C), which indicates that the process is more favorable at higher temperatures. The positive ΔS^0^ value demonstrated the affinity of MB for HG-10 and MGH-10 hydrogels and an irregular increase in the randomness of the solid–liquid interface during the adsorption process [51]. Moreover, the positive ΔH^0^ value of the adsorbents HG-10 and MHG-10 reveals that the adsorption process is endothermic [14].

### 2.6. Reusability of Adsorbent

The purpose of performing the adsorption–desorption test is to evaluate the reusability of the MHG-10 adsorbent (Figure 10b). Adsorption of the first cycle was performed with 10 mg of sample in 10 mL of 50 mg/L MB solution, pH = 6.8, for 10 min. Desorption was performed by immersing the magnetic hydrogel in 0.01 M HCl since the interaction between MB and MHG-10 is weakened by the presence of H^+^, causing desorption of MB. For the second adsorption cycle, the magnetic hydrogel was previously washed with a 0.01 M NaOH solution, and then MB (50 mg/L) was added under the same pH, time, and temperature conditions as the first cycle. The third cycle was performed analogously to the second cycle. The results show that the magnetic hydrogel MHG-10 can be used for four cycles while maintaining a removal percentage of 90%. In the fifth cycle, the removal percentage decreases to 69%. Therefore, the prepared MHG-10 can be used as a recyclable adsorbent for the removal of MB from wastewater.

## 3. Conclusions

In this work, magnetic hydrogels were prepared by in situ synthesis of Fe_3_O_4_ in p(HEMA-co-IA) hydrogels. The impact of Fe_3_O_4_ particles on the swelling behavior and adsorption capacity of MB was studied. The results obtained show that the amount of Fe_3_O_4_ particles is directly related to the amount of IA in the polymer chains. The degree of swelling in the magnetic hydrogels was higher compared to the hydrogels due to the repulsion of the carboxylate groups (–COO^−^) and the affinity of the Fe_3_O_4_ particles for water. It was also found that the adsorption capacity of the MHG-10 magnetic hydrogel (174.997 mg/g) for the removal of MB was higher than that of the HG-10 hydrogel (78.850 mg/g) due to the electrostatic interaction of the carboxylate groups (–COO^−^) and the positive charge of MB. In addition, the ability of Fe_3_O_4_ particles to adsorb dyes on their surface through van der Waals attractive forces contributes positively to the adsorption of methylene blue. The adsorption process of MHG-10 and HG-10 is described by the pseudo-second-order model and the Langmuir adsorption isotherm. The thermodynamic studies show that the adsorption process is spontaneous and endothermic. The high percentage removal of the magnetic hydrogel (91% of MB 200 mg/L) within a few minutes and the adsorption–desorption results demonstrate the potential of this material for the removal of the cationic dye methylene blue.

## 4. Materials and Methods

### 4.1. Chemicals

2-hydroxyethylmethacrylate (HEMA) and itaconic acid (IA) as monomers, N,N’-methylenebisacrylamide (MBA) as crosslinker, ammonium persulfate (APS) as initiator, and N,N,N’,N’-Tetramethylethylenediamine (TEMED) as activator were acquired from Sigma-Aldrich Darmstadt, Germany and used without any further purification. Sodium hydroxide (NaOH) (Eka chemicals, Marietta, GA, USA.) and chloride acid (HCl) (RPE-ACS, Bernolsheim, France) were used as received. Ferrous chloride tetrahydrate (FeCl_2_.4H_2_O) and ferric chloride hexahydrate (FeCl_3_.6H_2_O) were of analytical grade and purchased from Merck, Darmstadt, Germany. The solvents ethanol (Spectrum-Chemical, New Brunswick, NJ, USA.) and water were bidistilled before use. Methylene blue was purchased from Merck, Darmstadt, Germany.

### 4.2. Synthesis of the Hydrogels (HG)

The poly(HEMA-co-IA) hydrogels were prepared through free radical polymerization of HEMA, IA, and MBA according to the method described by Huaman et al. [64]. Briefly, HEMA, IA, MBA, and APS were dissolved in the solvent water:ethanol 1:1. The mixture was stirred for 5 min, and then TEMED was added. This solution was immediately transferred to the cylindrical mold. The co-polymerization was carried out at RT for 24 h. At the end of the co-polymerization, the hydrogels were taken out of the mold and cut into circular disks of 3 × 12 mm. The disks were immersed in distilled water and kept at RT for 48 h. In order to remove unreacted compounds, the water was changed with fresh distilled water every 12 h. After 48 h, the disks were dried at RT for 15 days and stored for later use. The feed compositions used to synthesize the hydrogels are given in Table 6.

### 4.3. Synthesis of the Magnetic Hydrogels (MHGs)

The magnetic hydrogels were prepared by in situ synthesis of magnetic nanoparticles (Fe_3_O_4_) into poly(HEMA-co-IA) hydrogels via a co-precipitation reaction of FeCl_2_·4H_2_O and FeCl_3_·6H_2_O in the presence of NaOH. The procedure described by Sivudu and Rhee was followed with some modifications [34]. The HG disks swelled in water for 24 h, then were removed and immersed for 4 h in a 200 mL mixed solution of Fe^2+^ and Fe^3+^ ions in a molar ratio of 1:2 (the weight of the iron salts was equal to that of the dry hydrogels used). The disks loaded with Fe^2+^ and Fe^3+^ ions were removed from the mixed solution, washed with water, immersed in a 200 mL solution of NaOH 0.5 mol/L, and left overnight. The resulting disk-shaped magnetic hydrogels were removed from the NaOH solution, washed several times with water, and finally allowed to dry at RT for 20 days (until the weight was constant).

### 4.4. Instrumental Characterization

Attenuated Total Reflectance Fourier Transform Infrared (ATR-FTIR) spectra of the hydrogels were recorded on a Nicolet 380 spectrometer. A portion of the hydrogel was dried at 70 °C for 2 h, ground to a powder, and placed on the ATR glass. Spectra were recorded at RT in the 4000–400 cm^−1^ wavenumber range with a resolution of 4 cm^−1^ and 32 scans. Thermogravimetric analysis (TGA) of the hydrogels was performed using a Perkin Elmer STA6000 instrument (Valencia, CA, USA.). Dry hydrogels were used, and the analysis conditions were a heating rate of 5 °C/min from 30 to 600 °C in nitrogen atmosphere with a flow rate of 20 mL/min. The initial weight of each hydrogel was approximately 10 mg. The hydrogels were also analyzed by X-ray diffraction (XRD) using a BRUKER D8-ADVANCE diffractometer (Cu radiation, λ = 1.5406 Å). Nitrogen adsorption–desorption isotherms of the hydrogels were obtained using a Gemini VII 2390t. For this purpose, a quantity of the sample was degassed at 90 °C for 4 h, and the test temperature was 77 K. A vibrating sample magnetometer (VSM, Lima, Peru) was used to study the magnetic property of the magnetic hydrogel. The morphology of the hydrogels was observed using a Zeiss EVO-MA10 scanning electron microscope (SEM, Dublin, CA, USA.). For SEM measurements, the samples were covered with carbon.

### 4.5. Study of Swelling

The degree of swelling (q g/g) was determined gravimetrically. The dried non-magnetic and magnetic hydrogels were immersed in bidistilled water at RT for 24 h. Then, the swollen hydrogels were removed from the water, dried on the surface with tissue paper, and finally weighed. The degree of swelling was calculated with the following Equation (11):

(11) $$q=\frac{W_{s}-{\;W}_{d}}{W_{d}}$$

where W_s_ is the mass (g) of the swollen hydrogel, and W_d_ is the mass (g) of the dry hydrogel.

In order to study the pH response of hydrogels, swelling experiments were carried out in 0.01 mol/L NaOH and 0.01 mol/L HCl solutions.

### 4.6. Point of Zero Charge (PZC)

To determine the surface charge or point of zero charge (Pzc), 10 mg of hydrogels were dispersed in 10 mL of NaCl solution (0.1 M) with initial pH values ranging from 2 to 11. The final pH was then measured after 24 h of stirring. The ΔpH = (initial pH − final pH) was plotted against the initial pH to obtain the pHzpc (ΔpH = 0) [51].

### 4.7. Adsorption of MB

The adsorption experiments were performed as a function of four factors: the effect of pH, dose, contact time, and MB initial concentration. The typical procedure consists of mixing the hydrogels (previously, dry hydrogels were swollen in distilled water for 24 h) and 10 mL of MB aqueous solution. The concentration of MB aqueous solution was determined with the UV–VIS spectrophotometer at λ = 664 nm, and the absorbance was converted to concentration using linear regression curve obtained from the calibration curve (absorbance versus standard concentration of MB 2.5, 5.0, 7.5, and 10 mg/L). The removal efficiency (% R) and adsorption capacity (Qe) of the hydrogels were determined with Equations (12) and (13) [65]:

(12) $$\%\;R\;=\left(\frac{C_{0\;}-C_{e}}{C_{0}}\right)\times 100$$

(13) $$Q_{e}=\frac{(C_{0}-C_{e})V}{m}\;$$

where C_0_ is the initial concentration of MB, C_e_ is the concentration of MB at equilibrium (mg/L), V is the volume (L) of MB solution, and m is the mass (g) of dried hydrogel.

To investigate the effect of pH on MB removal, the hydrogels were mixed with 10 mL of 50 mg/L MB aqueous solution at pH 2–10. The effect of adsorbent dosage was carried out with 5–50 mg of sample, while the amount of MB aqueous solution was fixed in each experiment. For contact time, the hydrogels were mixed with 10 mL of 50 mg/L MB aqueous solution, and the MB concentration was determined after 10–150 min. The effect of initial concentration was evaluated with 10 mL aqueous MB solutions at 50–200 mg/L.

### 4.8. Reuse Studies

The reusability of the magnetic hydrogel was investigated by performing desorption studies. The experiments were carried out as follows: 10 mg of magnetic hydrogel was swollen in distilled water for 24 h, then the water was removed and MB solution (50 mg/L) with pH = 6.8 was added and kept in contact for 10 min. For the desorption process, the magnetic hydrogel loaded with the MB dye was contacted with 10 mL of 0.01 M HCl solution for 1 h to remove the adsorbed MB dye. Before performing the second adsorption cycle under the same conditions as the first cycle, the magnetic hydrogel was washed with a 0.01 M NaOH solution.

## Figures and Tables

**Table 1:** Swelling degree (g/g) of non-magnetic and magnetic hydrogels after 24 h immersion in different media.

Amount of IA (mol%)	Water q (g/g)		NaOH 0.01 mol/L q (g/g)		HCl 0.01 mol/L q (g/g)	
	HG	MHG	HG	MHG	HG	MHG
0	0.723	24.587	0.940	15.024	0.812	0.801
5	10.524	52.937	18.290	27.459	0.732	0.766
7.5	14.290	54.631	23.037	28.406	0.824	0.969
10	12.915	42.310	31.535	31.307	0.870	0.846

**Table 2:** Kinetic parameters for MB removal using HG-10 and MHG-10.

		Adsorbent	
Kinetic Models	Parameters	HG-10	MHG-10
	Q_e_ (cal) (mg/g)	0.529	0.171
Pseudo-first-order	K_1_ (1/min)	0.035	0.024
	R^2^	0.984	0.978
	Q_e_ (exp) (mg/g)	24.626	49.709
Pseudo-second-order	Q_e_ (cal) (mg/g)	24.652	49.718
	K_2_ (g/(mg·min))	0.181	0.412
	R^2^	0.999	0.999
	Q_e_ (exp) (mg/g)	24.626	49.709
Intraparticle diffusion	KD	0.0411	0.0153
	c	24.188	49.536
	R^2^	0.7928	0.8987

**Table 3:** Isothermal parameters for MB removal using HG-10 and MHG-10.

		Adsorbent	
Isotherm	Parameters	HG-10	MHG-10
	Q_max_ (mg/g)	78.850	174.997
Langmuir	K_L_ (L/mg)	0.399	1.082
	R^2^	0.999	0.972
	R_L_	0.0477–0.0124	0.0182–0.0046
Freundlich	K_F_ (mg/g)	28.826	86.605
	1/n	0.292	0.256
	R^2^	0.906	0.845
Temkin	K_T_ (L/g)	7.295	31.467
	b_T_ (J/mol)	177.815	90.335
	R^2^	0.952	0.907
D–R	q_s_ (mg/g)	59.969	144.510
	E (kJ/mol)	1.242	2.350
	R^2^	0.818	0.900

**Table 4:** Comparison of the maximum adsorption capacity of MHG-10 magnetic hydrogel and other adsorbents for the removal of MB.

Adsorbent	Q (mg/g)	Reference
Hydrogel sodium alginate/Fe_3_O_4_	186.57	[57]
Fe_3_O_4_ nanoparticles	91.9	[58]
Poly(AAB) magnetic hydrogel	12.64	[59]
Magnetic chitosan microspheres (TETA-MCTSms)	211.22	[60]
Fe_3_O_4_@SiO_2_ magnetic nanoparticles	37.52	[61]
NH_2_-MWCNTs@Fe_3_O_4_	178.5	[62]
P(NIPAAm/IA/pumice) hydrogel	22.62	[63]
Poly(HEMA-co-IA) magnetic hydrogel (MHG-10)	174.99	This work

**Table 5:** Thermodynamic parameters for MB adsorption using MHG-10 and HG-10.

Adsorbent	T °C	ΔG^0^ (kJ/mol)	ΔH^0^ (kJ/mol)	ΔS^0^ (J/mol·K)
MHG-10	25	−2.764	30.195	110.544
	35	−3.869		
	45	−4.974		
HG-10	25	−3.124	34.131	124.954
	35	−4.373		
	45	−5.623		

**Table 6:** Chemical compositions for the synthesis of hydrogels (HGs).

	Hydrogels HG ^a^ Poly(HEMA-co-IA) *			
	HEMA g (mol%)	IA g (mol%)	MBA g (mol%)	APS g (mol%)
HG-0 ^b^	4.00 (100)	0.00 (0)	0.24 (5)	0.07 (1)
HG-5	3.80 (95)	0.20 (5)	0.24 (5)	0.07 (1)
HG-7.5	3.70 (92.5)	0.30 (7.5)	0.24 (5)	0.07 (1)
HG-10	3.60 (90)	0.40 (10)	0.24 (5)	0.07 (1)

* In each experiment, 12 drops of TEMED and 20 mL of EtOH-H2O 1:1 solvent were used. ^a^ In the HG, the number after the hyphen represents the percentage (mol%) of IA. ^b^ In this case, 4.00 g of HEMA represents 100 mol%, from which the other calculations are performed as indicated in the table.

## Data Availability

The data presented in this study are openly available in article.

## References

[1] Anzules Í.d.C.P., Castro D.W.M. (2022). Contaminación ambiental. Recimundo.

[2] Karapanagioti H.K., Kalavrouziotis I.K. (2022). Microplastics in Water Bodies and in the Environment. Water.

[3] Zhou Q., Yang N., Li Y., Ren B., Ding X., Bian H., Yao X. (2020). Total concentrations and sources of heavy metal pollution in global river and lake water bodies from 1972 to 2017. Glob. Ecol. Conserv..

[4] Islam T., Repon M.R., Islam T., Sarwar Z., Rahman M.M. (2022). Impact of textile dyes on health and ecosystem: A review of structure, causes, and potential solutions. Environ. Sci. Pollut. Res..

[5] Chen X., Memon H.A., Wang Y., Marriam I., Tebyetekerwa M. (2021). Circular Economy and Sustainability of the Clothing and Textile Industry. Mater. Circ. Econ..

[6] Khattab T.A., Abdelrahman M.S., Rehan M. (2020). Textile dyeing industry: Environmental impacts and remediation. Environ. Sci. Pollut. Res..

[7] Naushad M., Alqadami A.A., AlOthman Z.A., Alsohaimi I.H., Algamdi M.S., Aldawsari A.M. (2019). Adsorption kinetics, isotherm and reusability studies for the removal of cationic dye from aqueous medium using arginine modified activated carbon. J. Mol. Liq..

[8] Khan I., Saeed K., Zekker I., Zhang B., Hendi A.H., Ahmad A., Ahmad S., Zada N., Ahmad H., Shah L.A. (2022). Review on Methylene Blue: Its Properties, Uses, Toxicity and Photodegradation. Water.

[9] Oladoye P.O., Ajiboye T.O., Omotola E.O., Oyewola O.J. (2022). Methylene blue dye: Toxicity and potential elimination technology from wastewater. Results Eng..

[10] Ihaddaden S., Aberkane D., Boukerroui A., Robert D. (2022). Removal of methylene blue (basic dye) by coagulation-flocculation with biomaterials (bentonite and *Opuntia ficus* indica). J. Water Process Eng..

[11] Moradihamedani P. (2022). Recent advances in dye removal from wastewater by membrane technology: A review. Polym. Bull..

[12] Saeed M., Muneer M., Haq A.U., Akram N. (2021). Photocatalysis: An effective tool for photodegradation of dyes—A review. Environ. Sci. Pollut. Res..

[13] Goswami K., Ulaganambi M., Sukumaran L.K., Tetala K.K.R. (2023). Synthesis and application of iron based metal organic framework for efficient adsorption of azo dyes from textile industry samples. Adv. Sample Prep..

[14] Mittal H., Al Alili A., Morajkar P.P., Alhassan S.M. (2021). Graphene oxide crosslinked hydrogel nanocomposites of xanthan gum for the adsorption of crystal violet dye. J. Mol. Liq..

[15] Sultana M., Rownok M.H., Sabrin M., Rahaman M.H., Alam S.M.N. (2022). A review on experimental chemically modified activated carbon to enhance dye and heavy metals adsorption. Clean. Eng. Technol..

[16] Harja M., Lupu N., Chiriac H., Herea D.D., Buema G. (2022). Studies on the Removal of Congo Red Dye by an Adsorbent Based on Fly-Ash@Fe_3_O_4_ Mixture. Magnetochemistry.

[17] Wang G., Li G., Huan Y., Hao C., Chen W. (2020). Acrylic acid functionalized graphene oxide: High-efficient removal of cationic dyes from wastewater and exploration on adsorption mechanism. Chemosphere.

[18] Subhan F., Aslam S., Yan Z., Yaseen M., Naeem M., Ikram M., Ali A., Bibi S. (2022). Adsorption and reusability performance of hierarchically porous silica (MMZ) for the removal of MB dye from water. Inorg. Chem. Commun..

[19] Guo S., Xu H., Zhang F., Zhu X., Li X. (2018). Preparation and adsorption properties of nano magnetite silica gel for methylene blue from aqueous solution. Colloids Surf. A Physicochem. Eng. Asp..

[20] Andolsi A., Chaari I., Hamzaoui A.H. (2023). Surface Modification of Magnetite Using Silica Coating: Spectroscopic, Structural, Morphological Characterization and Interaction with Crystal Violet Dye. Silicon.

[21] Imessaoudene A., Cheikh S., Hadadi A., Hamri N., Bollinger J.C., Amrane A., Tahraoui H., Manseri A., Mouni L. (2023). Adsorption Performance of Zeolite for the Removal of Congo Red Dye: Factorial Design Experiments, Kinetic, and Equilibrium Studies. Separations.

[22] Rakanović M., Vukojević A., Savanović M.M., Armaković S., Pelemiš S., Živić F., Sladojević S., Armaković S.J. (2022). Zeolites as Adsorbents and Photocatalysts for Removal of Dyes from the Aqueous Environment. Molecules.

[23] Shi T., Xie Z., Mo X., Feng Y., Peng T., Song D. (2022). Highly Efficient Adsorption of Heavy Metals and Cationic Dyes by Smart Functionalized Sodium Alginate Hydrogels. Gels.

[24] Mouhtady O., Obeid E., Abu-samha M., Younes K., Murshid N. (2022). Evaluation of the Adsorption Efficiency of Graphene Oxide Hydrogels in Wastewater Dye Removal: Application of Principal Component Analysis. Gels.

[25] Darban Z., Shahabuddin S., Gaur R., Ahmad I., Sridewi N. (2022). Hydrogel-Based Adsorbent Material for the Effective Removal of Heavy Metals from Wastewater: A Comprehensive Review. Gels.

[26] Kaniewska K., Karbarz M., Katz E. (2020). Nanocomposite hydrogel films and coatings—Features and applications. Appl. Mater. Today.

[27] Yao G., Bi W., Liu H. (2020). pH-responsive magnetic graphene oxide/poly(NVI-co-AA) hydrogel as an easily recyclable adsorbent for cationic and anionic dyes. Colloids Surf. A Physicochem. Eng. Asp..

[28] Zhang C., Dai Y., Wu Y., Lu G., Cao Z., Cheng J., Wang K., Yang H., Xia Y., Wen X. (2020). Facile preparation of polyacrylamide/chitosan/Fe_3_O_4_ composite hydrogels for effective removal of methylene blue from aqueous solution. Carbohydr. Polym..

[29] Hosseini S.S., Hamadi A., Foroutan R., Peighambardoust S.J., Ramavandi B. (2022). Decontamination of Cd^2+^ and Pb^2+^ from aqueous solution using a magnetic nanocomposite of eggshell/starch/Fe_3_O_4_. J. Water Process Eng..

[30] Mehdizadeh A., Moghadam P.N., Ehsanimehr S., Fareghi A.R. (2022). Preparation of a New Magnetic Nanocomposite for the Removal of Dye Pollutions from Aqueous Solutions: Synthesis and Characterization. Mater. Chem. Horiz..

[31] Beigi P., Ganjali F., Hassanzadeh-Afruzi F., Salehi M.M., Maleki A. (2023). Enhancement of adsorption efficiency of crystal violet and chlorpyrifos onto pectin hydrogel@Fe3O4-bentonite as a versatile nanoadsorbent. Sci. Rep..

[32] Singh N., Yadav S., Mehta S.K., Dan A. (2022). In situ incorporation of magnetic nanoparticles within the carboxymethyl cellulose hydrogels enables dye removal. J. Macromol. Sci. Part A.

[33] Filho E., Brito E., Silva R., Streck L., Bohn F., Fonseca J. (2021). Superparamagnetic polyacrylamide/magnetite composite gels. J. Dispers. Sci. Technol..

[34] Sivudu K.S., Rhee K.Y. (2009). Preparation and characterization of pH-responsive hydrogel magnetite nanocomposite. Colloids Surf. A Physicochem. Eng. Asp..

[35] Shen X.C., Fang X.Z., Zhou Y.H., Liang H. (2004). Synthesis and Characterization of 3-Aminopropyltriethoxysilane-Modified Superparamagnetic Magnetite Nanoparticles. Chem. Lett..

[36] Petcharoen K., Sirivat A. (2012). Synthesis and characterization of magnetite nanoparticles via the chemical co-precipitation method. Mater. Sci. Eng. B.

[37] Samrot A.V., Ali H.H., Selvarani A.J., Faradjeva E., Raji P., Prakash P., Kumar S.S. (2021). Adsorption efficiency of chemically synthesized Superparamagnetic Iron Oxide Nanoparticles (SPIONs) on crystal violet dye. Curr. Res. Green. Sustain. Chem..

[38] Taleb M.F.A., El Fadl F.I.A., Albalwi H. (2021). Adsorption of toxic dye in wastewater onto magnetic NVP/CS nanocomposite hydrogels synthesized using gamma radiation. Sep. Purif. Technol..

[39] Cai J., Guo J., Ji M., Yang W., Wang C., Fu S. (2007). Preparation and characterization of multiresponsive polymer composite microspheres with core-shell structure. Colloid. Polym. Sci..

[40] Jiang Y., Cai W., Tu W., Zhu M. (2019). Facile Cross-Link Method to Synthesize Magnetic Fe_3_O_4_@SiO_2_-Chitosan with High Adsorption Capacity toward Hexavalent Chromium. J. Chem. Eng. Data.

[41] Thommes M., Kaneko K., Neimark A.V., Olivier J.P., Rodriguez-Reinoso F., Rouquerol J., Sing K.S.W. (2015). Physisorption of gases, with special reference to the evaluation of surface area and pore size distribution (IUPAC Technical Report). Pure Appl. Chem..

[42] Zhang W., Lan Y., Ma M., Chai S., Zuo Q., Kim K.H., Gao Y. (2020). A novel chitosan–vanadium-titanium-magnetite composite as a superior adsorbent for organic dyes in wastewater. Environ. Int..

[43] Yue J., Wang Z., Chen J., Zheng M., Wang Q., Lou X. (2019). Investigation of pore structure characteristics and adsorption characteristics of coals with different destruction types. Adsorpt. Sci. Technol..

[44] Li X., Gao Z., Fang S., Ren C., Yang K., Wang F. (2019). Fractal Characterization of Nanopore Structure in Shale, Tight Sandstone and Mudstone from the Ordos Basin of China Using Nitrogen Adsorption. Energies.

[45] Qi L., Tang X., Wang Z., Peng X. (2017). Pore characterization of different types of coal from coal and gas outburst disaster sites using low temperature nitrogen adsorption approach. Int. J. Min. Sci. Technol..

[46] Ozay O., Ekici S., Baran Y., Aktas N., Sahiner N. (2009). Removal of toxic metal ions with magnetic hydrogels. Water Res..

[47] Santillán F., Rueda J.C. (2020). Removal of Methylene Blue by Hydrogels based on N, N-Dimethylacrylamide and 2-Oxazoline macromonomer. J. Polym. Res..

[48] Panda S.K., Aggarwal I., Kumar H., Prasad L., Kumar A., Sharma A., Vo D.V.N., Van Thuan D., Mishra V. (2021). Magnetite nanoparticles as sorbents for dye removal: A review. Environ. Chem. Lett..

[49] Zhao L.X., Xiao H., Li M.H., Xie M., Li N., Zhao R.S. (2021). Effectively removing indole-3-butyric acid from aqueous solution with magnetic layered double hydroxide-based adsorbents. J. Hazard. Mater..

[50] Foroutan R., Mohammadi R., Ahmadi A., Bikhabar G., Babaei F., Ramavandi B. (2022). Impact of ZnO and Fe3O4 magnetic nanoscale on the methyl violet 2B removal efficiency of the activated carbon oak wood. Chemosphere.

[51] Chen L., Zhu Y., Cui Y., Dai R., Shan Z., Chen H. (2021). Fabrication of starch-based high-performance adsorptive hydrogels using a novel effective pretreatment and adsorption for cationic methylene blue dye: Behavior and mechanism. Chem. Eng. J..

[52] Bharathiraja B., Selvakumari I.A.E., Iyyappan J., Varjani S. (2019). Itaconic acid: An effective sorbent for removal of pollutants from dye industry effluents. Curr. Opin. Environ. Sci. Health.

[53] Onder A., Ilgin P., Ozay H., Ozay O. (2020). Removal of dye from aqueous medium with pH-sensitive poly[(2-(acryloyloxy)ethyl]trimethylammonium chloride-co-1-vinyl-2-pyrrolidone] cationic hydrogel. J. Environ. Chem. Eng..

[54] Noori M., Tahmasebpoor M., Foroutan R. (2022). Enhanced adsorption capacity of low-cost magnetic clinoptilolite powders/beads for the effective removal of methylene blue: Adsorption and desorption studies. Mater. Chem. Phys..

[55] Hu X.S., Liang R., Sun G. (2018). Super-adsorbent hydrogel for removal of methylene blue dye from aqueous solution. J. Mater. Chem. A Mater..

[56] Safarzadeh H., Peighambardoust S.J., Mousavi S.H., Foroutan R., Mohammadi R., Peighambardoust S.H. (2022). Adsorption ability evaluation of the poly(methacrylic acid-co-acrylamide)/cloisite 30B nanocomposite hydrogel as a new adsorbent for cationic dye removal. Environ. Res..

[57] Wu Z., Wu J., Huang M., Liang H., Sun B. (2024). Preparation of reusable hydrogel spheres based on sodium alginate/Fe_3_O_4_ modified with carboxymethyl Huangshui polysaccharide and the efficient adsorption performance for methylene blue. Food Chem..

[58] Ghaedi M., Hajjati S., Mahmudi Z., Tyagi I., Agarwal S., Maity A., Gupta V.K. (2015). Modeling of competitive ultrasonic assisted removal of the dyes—Methylene blue and Safranin-O using Fe_3_O_4_ nanoparticles. Chem. Eng. J..

[59] Li S., Liu X., Huang W., Li W., Xia X., Yan S., Yu J. (2011). Magnetically assisted removal and separation of cationic dyes from aqueous solution by magnetic nanocomposite hydrogels. Polym. Adv. Technol..

[60] Yu S., Cui J., Jiang H., Zhong C., Meng J. (2019). Facile fabrication of functional chitosan microspheres and study on their effective cationic/anionic dyes removal from aqueous solution. Int. J. Biol. Macromol..

[61] Lei Y., Zhang X., Meng X., Wang Z. (2022). The preparation of core–shell Fe_3_O_4_@SiO_2_ magnetic nanoparticles with different surface carboxyl densities and their application in the removal of methylene blue. Inorg. Chem. Commun..

[62] Ahamad T., Naushad M., Eldesoky G.E., Al-Saeedi S.I., Nafady A., Al-Kadhi N.S., Al-Muhtaseb A.H., Khan A.A., Khan A. (2019). Effective and fast adsorptive removal of toxic cationic dye (MB) from aqueous medium using amino-functionalized magnetic multiwall carbon nanotubes. J. Mol. Liq..

[63] Taşdelen B., Çifçi D.İ., Meriç S. (2017). Preparation of N-isopropylacrylamide/itaconic acid/Pumice highly swollen composite hydrogels to explore their removal capacity of methylene blue. Colloids Surf. A Physicochem. Eng. Asp..

[64] Huaman M.A.L., Vega-Chacón J., Quispe R.I.H., Negrón A.C.V. (2023). Synthesis and swelling behaviors of poly(2-hydroxyethyl methacrylate-co-itaconic acid) and poly(2-hydroxyethyl methacrylate-co-sodium itaconate) hydrogels as potential drug carriers. Results Chem..

[65] Ahmadi A., Foroutan R., Esmaeili H., Peighambardoust S.J., Hemmati S., Ramavandi B. (2022). Montmorillonite clay/starch/CoFe_2_O_4_ nanocomposite as a superior functional material for uptake of cationic dye molecules from water and wastewater. Mater. Chem. Phys..

